# Stressors and level of stress among different nursing positions and the associations with hyperlipidemia, hyperglycemia, and hypertension: a national questionnaire survey

**DOI:** 10.1186/s12912-021-00777-y

**Published:** 2021-12-13

**Authors:** Po-Ya Chang, Shu-Ti Chiou, Wen-Yen Lo, Nicole Huang, Li-Yin Chien

**Affiliations:** 1 Department of Surgery, National Taiwan University Hospital, Taipei, Taiwan; 2 Institute of Public Health, National Yang Ming Chaio Tung University, Yang-Ming Campus, Taipei, Taiwan; 3 Center for Healthcare Quality Management, Cheng Hsin General Hospital, Taipei, Taiwan; 4 Department of Nursing, National Yang Ming Chiao Tung University, Yang-Ming Campus, Taipei, Taiwan; 5 Institute of Hospital and Health Care Administration, National Yang Ming Chiao Tung University, Yang-Ming Campus, Taipei, Taiwan; 6 Institute of Community Health Care, College of Nursing, National Yang Ming Chiao Tung University, Yang-Ming Campus, 155 Li-Nong Street, Section 2, Bei-Tou, Taipei, 11221 Taiwan

**Keywords:** Hypertension, Hyperglycemia, Hyperlipidemia, Nurses, Nursing, supervisory, Head nurses, Nurse practitioners, Job stress

## Abstract

**Background:**

Nurses are faced with varying job stressors depending on their positions and duties. Few previous studies have compared job stress and related chronic conditions among different nursing positions. The objectives were to compare job stressors among clinical registered nurses, nurse practitioners, and head nurses and explore the impact of job stressors and stress level on hyperlipidemia, hyperglycemia, and hypertension.

**Methods:**

Secondary data extracted from a survey of health-care workers conducted from May to July 2014 across 113 hospitals in Taiwan was used. This analysis included 17,152 clinical registered nurses, 1438 nurse practitioners, and 2406 head nurses. Socio-demographic characteristics, job stressors, stress levels, and hyperlipidemia, hyperglycemia, and hypertension variables were extracted.

**Results:**

Perceived stressors differed among clinical registered nurses, nurse practitioners, and head nurses, but overall stress level did not. Nurse practitioners and head nurses showed significantly higher prevalence of hyperlipidemia, hyperglycemia, and hypertension than clinical registered nurses. Higher stress levels, age, body mass index, work hours, and caring for family members were positively associated with hyperlipidemia, hyperglycemia, and hypertension. After adjustment for these variables, risk of hyperlipidemia, hyperglycemia, and hypertension did not differ across the nursing positions.

**Conclusions:**

Although stressors vary by different nursing positions, overall stress level does not. Hyperlipidemia, hyperglycemia, and hypertension are related to stress level, age, body mass index, weekly working hours, and caring for family members. Hence, alleviating job stress and avoiding long working hours are likely to reduce the risk of hyperlipidemia, hyperglycemia, and hypertension in nurses.

## Introduction

In many countries, nursing is considered as a highly stressful occupation [1], and high work stress is a long-term problem in the health-care industry [1, 2]. Studies have found work stress to be related to hyperlipidemia, hyperglycemia, and hypertension [3–5]; however, the strength of these associations across different nursing positions has not been well-established. In the health-care system, nursing staff perform various roles and functions, with their specific responsibilities depending on their positions [6]; thus, the stressors and work stress experienced by nurses may differ depending on their job responsibilities.

Encountering stress from various sources at work may cause distress symptoms, and inadequate treatment of these symptoms could result in the development of physiological and/or mental diseases [7]. Over the past 20 years, many studies have examined the correlation between work stress and hyperlipidemia, hyperglycemia, and hypertension [3, 5], as well as the possibility of such stress increasing the risk of coronary heart disease [8].

Several cross-sectional studies have reported that work stress is a risk factor for hyperlipidemia [3, 9, 10]. Several studies, including laboratory data papers [11], case-control studies [12], and meta-analyses [4, 13] have shown that stress increases blood glucose levels. For example, Kivimäki and Kawachi (2015) examined 27 cohort studies on work stress and type 2 diabetes and found that high work stress is a risk factor for type 2 diabetes [4]. Systematic reviews and meta-analyses have shown that work stress is related to hypertension [5, 14, 15]. Notably, Landsbergis (2013) examined 22 cross-sectional studies and found work stress to be significantly related to hypertension [14]. However, despite the above findings, the association between job stress and conditions of hyperlipidemia, hyperglycemia, and hypertension has not been examined and compared across different nursing positions.

Nurses account for a large proportion of the personnel in the health-care industry. They play a crucial role in clinical care, and have a wide variety of responsibilities [16]. However, nurses were found to experience moderate to high work stress [17, 18], with their work stressors including workloads, shifts, role conflicts, interpersonal relationships, workplace violence, insufficient preparation, and uncertainty regarding treatment results [1, 2, 19].

The United States (US)’ health-care system features four types of advanced-practice registered nurses: certified nurse practitioners (NPs), certified registered nurse anesthetists, clinical nurse specialists, and certified nurse midwives [20]. NPs’ primary responsibilities include patient care, health education, medical coordination, and medical quality control [20]. In the Taiwanese medical system, clinical nursing staff can be divided into clinical registered nurses (CNs), NPs, and head nurses/nurse managers (HNs). In Taiwan, approximately 83% of nurses work in hospitals [21], with NPs performing much of the clinical work in hospitals; in 2018, there were 7685 practicing NPs in Taiwan [22]. NPs, who are supervised by physicians, were found to experience moderate work stress, originating from factors such as the CN to NP transition, low confidence, the need to meet the expectations of team members, and anxiety regarding the opinions of colleagues [23–25]. Meanwhile, HNs are responsible for the general operation of the entire ward and quality maintenance of patient care in the unit; HNs also commonly experience considerable stress during work [26], with one in six HNs experiencing work stress and job burnout [27]. Stressors for HNs include role conflicts, time pressure, workload, insufficient workplace support, insufficient leadership, rapid transformations of medical systems, and organizational limitations [16, 26].

Previous studies on work stress among nurses have compared job stress across hospital levels, work units, and qualifications [16, 28]; however, few studies have examined the level of perceived job-related stress, and the association of such stress with diseases, across different job positions. Therefore, this study aimed to compare job stressors among CNs, NPs, and HNs, and examine the impact of different job stressors and overall level of stress on the prevalence of hyperlipidemia, hyperglycemia, and hypertension among these nurses.

## Methods

### Design and participants

This secondary data analysis was performed using data sourced from a national cross-sectional study of full-time health-care workers in Taiwan that was conducted from July to September 2014. This study invited those health facilities that ever jointed the Taiwan Health Promoting Hospital Network, ever participated in the previous similar survey in 2011, or ever applied for certification of health promoting hospital and health-care institution to participate. Of the 157 health facilities invited, 113 agreed to participate, including 13 medical centers, 59 district hospitals, 32 regional hospitals, eight public-health centers, and one nursing home. After obtaining approval from the hospitals, envelopes with consent forms and questionnaires were distributed to each of the hospital. Health-care workers were asked to return the signed consent forms and filled questionnaires using the provided two separated and sealed envelopes to the data collection site at each hospital. Overall, 111,110 questionnaires were distributed, 89,014 filled questionnaires were returned, and 75,841 valid questionnaires were collected (valid response rate = 68.3%). The dataset with valid records were released to the research team by the Taiwan Health Promotion Administration. For the present study, questionnaires answered by non-nursing staff (physicians, pharmacists, and other medical professionals) and non-clinical staff (administrative staff, medical laboratory personnel, laborer, and others) were excluded, remaining 20,996 anonymous questionnaires completed by full-time clinical nurses, which formed the analytical sample for this secondary data analysis. Among these nurses, 2406 (11.5%) were HNs, 1438 (6.8%) were NPs, and 17,152 (81.7%) were CNs. Details of the study design are presented elsewhere [29].

### Measures

Data were collected through a structured questionnaire that focused on the physical and mental health, and safety of the participants. Prior to the data collection, six experts reviewed and revised the questionnaire content over two meetings, and a pilot test was performed on 10 health-care workers to ensure its clarity and readability.

Hyperlipidemia, hyperglycemia, and hypertension were measured using yes/no questions regarding the presence of any of those chronic conditions. The study variables related to hyperlipidemia, hyperglycemia, and hypertension included socio-demographics (age, gender, and marital status), work experience (years of work experience, work unit, job position), medical history and chronic diseases, self-reported weight and height, and family-care needs. Family-care needs was measured by a question enquiring “current family-care needs” with four items -- (1) self or spouse being pregnant, (2) having children at preschool age, (3) having children in elementary school and/or high school, (4) having other family member who is in need of care. Any positive answer to the four items was coded as yes, otherwise it was coded as no.

Sources of work stress and level of perceived stress were measured by a scale of 23 items. A five-point Likert scale (1–5) was used to measure level of perceived stress; higher scores indicated greater stress, given a summary score ranged from 23 to 115 points. The reliability and validity of the scale have been described previously [29]. The scale explained 62.13% of the variance, and the internal consistency was 0.93 [18], supporting construct validity and reliability of the scale. Meanwhile, the 23 items represented eight potential sources of job stress: (1) evaluation and policy; (2) colleagues, higher-level officers, and extra-occupational duties; (3) supervisor of own department; (4) work content and opinions of individuals from other departments; (5) lack of work leadership, security, and equality; (6) impact of and fatigue from work performance; (7) promotion; and (8) others.

### Data analysis

Statistical analysis was performed using SPSS 23.0 (IBM Corp., Armonk, NY, USA). Means and standard deviations were used to describe continuous variables, and frequencies and percentages were used to describe categorical variables and the overall distribution of the sample. Analysis of variance (ANOVA) was used to compare the level of stress among the three different nurse positions. If a significant difference was found, the Scheffé post-hoc test was conducted for pairwise comparisons. Further, logistic regression models were applied to examine the factors associated with the presence of any of the chronic conditions including hyperlipidemia, hyperglycemia, and hypertension.

## Results

Table 1 shows the characteristics of the 20,996 nurses. HNs had the highest mean age (39.1 years, standard deviation [*SD*]: 7.09), followed by NPs (37.6, *SD*: 5.55), and CNs (31.7, *SD*: 0.66). Most participants (98%) were women. Over one quarter (26.2%) were overweight or obese (body mass index [BMI] > 24). Over half (60%) of the NPs and HNs were married; most CNs (> 60%) were never married. HNs had the highest mean number of years of work experience, at 20.35 (*SD*: 9.75), followed by NPs at 15.14 (*SD*: 9.72) and CNs at 11.96 (*SD*: 10.44). The general ward was the most-represented work unit (42.7%). HNs had the highest mean number of work hours per week (50.72, *SD*: 12.84), followed by NPs (49.98, *SD*: 12.18) and CNs (47.49, *SD*: 11.65). The CN group had the highest proportion of members who had family-care needs (49.6%).

**Table 1:** Characteristics of the participating nurses

Variables	All (*n* = 20,996) n (%)	Clinical nurses (*n* = 17,152) n (%)	Nurse practitioners (*n* = 1438) n (%)	Head nurses (*n* = 2406) n (%)	***p*** value
**Age** (years)					
< 26	4093 (20.4)	4062 (24.8)	15 (1.1)	16 (0.7)	< 0.001^*^
26–35	9107 (45.5)	7832 (47.9)	542 (39.6)	733 (31.8)	
36–45	5089 (25.4)	3328 (20.4)	704 (51.5)	1057 (45.9)	
46–55	1586 (7.9)	1017 (6.2)	102 (7.5)	467 (20.3)	
> 55	143 (0.7)	108 (0.7)	4 (0.3)	31 (1.3)	
**Gender**					
Male	412 (2.0)	311 (1.9)	65 (4.7)	36 (1.6)	< 0.001^*^
Female	19,883 (98.0)	16,279 (98.1)	1320 (95.3)	2284 (98.4)	
**BMI (**kg/m^2^)					
< 18.5	2124 (11.9)	1894 (12.9)	91 (7.6)	139 (6.8)	< 0.001^*^
18.5–23.9	11,077 (62.0)	9058 (61.9)	792 (65.7)	1227 (60.2)	
> 24	4678 (26.2)	3684 (25.2)	322 (26.7)	672 (33.0)	
**Marital status**					
Never married	10,796 (54.8)	9672 (60.1)	437 (32.3)	687 (30.3)	< 0.001^*^
Married	8390 (42.6)	6048 (37.6)	846 (62.6)	1496 (66.0)	
Divorced or widowed	516 (2.6)	365 (2.3)	68 (5.0)	83 (3.7)	
**Work experience** (years)					
< 5	6719 (35.5)	6286 (40.9)	303 (23.1)	130 (5.7)	< 0.001^*^
5–9	328 (1.7)	240 (1.6)	24 (1.8)	64 (2.8)	
10–14	3045 (16.1)	2206 (14.4)	284 (21.7)	555 (24.5)	
> 15	8852 (46.7)	6639 (43.2)	698 (53.3)	1515 (66.9)	
**Work unit**					
OPD	2584 (12.3)	2384 (13.9)	31 (2.2)	169 (7.0)	< 0.001^*^
ER/ICU	4736 (22.6)	3929 (22.9)	269 (18.7)	538 (22.4)	
General ward	8955 (42.7)	7174 (41.8)	692 (48.1)	1089 (45.3)	
OR/DR	2428 (11.6)	2114 (12.3)	69 (4.8)	245 (10.2)	
Multiple units	2293 (10.9)	1551 (9.0)	377 (26.2)	365 (15.2)	
**Weekly work hours**					
≤ 40	5260 (28.4)	4538 (30.1)	238 (18.9)	484 (22.5)	< 0.001^*^
41–49	8060 (43.6)	6622 (43.9)	590 (46.9)	848 (39.5)	
> 50	5184 (28.0)	3938 (26.1)	431 (34.2)	815 (38.0)	
**Family-care needs**	9889 (47.1)	8500 (49.6)	507 (35.3)	882 (36.7)	< 0.001^*^
**Any of the following**	1856 (9.5)	1358 (8.5)	163 (12.2)	335 (14.8)	< 0.001^*^
Hyperlipidemia	898 (4.6)	674 (4.2)	77 (5.8)	147 (6.5)	< 0.001^*^
Hyperglycemia	541 (2.8)	429 (2.7)	36 (2.7)	76 (3.3)	0.192
Hypertension	931 (4.8)	644 (4.0)	92 (6.9)	195 (8.6)	< 0.001^*^

*BMI* body mass index, *DR* delivery room, *ER* emergency room, *ICU* intensive care unit, *OPD* outpatient clinic, *OR* operating room

Multiple units indicated that participants worked in more than one of the listed units

Percentages were calculated based on the total number of non-missing cases

The prevalence among the sample of any of the constituents of hyperlipidemia, hyperglycemia, and hypertension was 9.5% (Table 1). Of these, most had hypertension (*n* = 931, 4.8%), followed by hyperlipidemia (*n* = 898, 4.6%) and hyperglycemia (*n* = 541, 2.8%). The prevalence of hyperlipidemia, hyperglycemia, and hypertension was highest among HNs (14.8%), followed by NPs (12.2%) and CNs (8.5%).

An ANOVA (Table 2) showed no significant difference among the nursing positions regarding scores for overall perceived work stress. However, analysis of stressors showed significant differences among the three nursing positions. The mean levels of evaluation- and policy-related stress were significantly higher among HNs than CNs and NPs. Meanwhile, scores for stress from colleagues, higher-level officers, and extra-occupational duties were significantly higher for HNs than NPs and CNs. NPs showed significantly higher scores for stress from work content and the opinions of individuals from other departments when compared to HNs and CNs. NPs showed the highest mean score for stress from inadequate work leadership, security, and equality, followed by CNs and HNs. Scores for stress from the impact of and fatigue from work performance were significantly higher among CNs and NPs than HNs. Promotion-induced stress was higher among CNs than among HNs and NPs. Finally, NPs showed higher mean scores for other sources of stress than did CNs and HNs.

**Table 2:** Sources of work stress for the different nursing positions; ***n*** **= 20,996**

Sources of stress	Clinical registered Nurses (*n* = 17,152) Mean (SD)	Nurse practitioners (*n* = 1438) Mean (SD)	Head Nurses (*n* = 2406) Mean (SD)	***p*** value	Post hoc	Aspects
**Overall level of work stress**	69.52 (15.53)	70.36 (14.51)	69.46 (15.42)	0.188	–	–
**Evaluation**	3.33 (1.16)	3.36 (1.14)	4.01 (1.07)	< 0.001	HN > CN = NP	1
**Health department or other external policy requirements**	3.17 (1.13)	3.09 (1.10)	3.71 (1.10)	< 0.001	HN > CN > NP	1
**Colleagues from own department**	2.70 (0.91)	2.71 (0.90)	2.79 (0.90)	< 0.001	HN > CN = NP	2
**Hospital management**	2.83 (1.05)	2.88 (1.03)	3.04 (1.03)	< 0.001	HN > CN = NP	2
**Too many extra-occupational duties**	3.36 (1.00)	3.31 (1.00)	3.46 (1.00)	< 0.001	HN > CN = NP	2
**Performance requirements**	2.94 (1.00)	2.94 (1.00)	3.09 (1.00)	< 0.001	HN > CN = NP	2
**Supervisor of own department**	2.98 (0.89)	3.04 (0.91)	3.04 (0.89)	< 0.001	NP = HN > CN	3
**Other departmental supervisors or colleagues**	2.54 (0.93)	2.63 (0.93)	2.58 (0.88)	0.008	NP > CN = HN	4
**Work difficulty and complexity**	3.24 (0.88)	3.32 (0.85)	3.25 (0.88)	< 0.001	NP > CN = HN	4
**Uncertainty regarding medical results or work outcomes**	3.26 (0.91)	3.36 (0.90)	3.26 (0.91)	< 0.001	NP > CN = HN	4
**Urgency of work**	3.13 (0.98)	3.19 (0.98)	3.13 (1,00)	0.003	NP > CN = HN	4
**Insufficient work leadership or support**	2.80 (0.93)	2.92 (0.95)	2.72 (0.93)	0.001	NP > CN > HN	5
**Lack of job security**	2.88 (1.03)	3.28 (1.04)	2.65 (1.01)	< 0.001	NP > CN > HN	5
**Unequal work requirements**	3.04 (1.05)	3.15 (1.04)	2.94 (1.05)	< 0.001	NP > CN > HN	5
**Patients**	3.13 (0.87)	3.10 (0.83)	2.82 (0.88)	< 0.001	CN = NP > HN	6
**Workload or working hours**	3.36 (0.89)	3.36 (0.87)	3.33 (0.90)	< 0.001	CN = NP > HN	6
**Labor burden**	3.33 (0.92)	3.31 (0.90)	3.25 (0.94)	< 0.001	CN = NP > HN	6
**Job hazards**	3.23 (0.95)	3.20 (0.92)	2.97 (0.95)	< 0.001	CN = NP > HN	6
**Contact with death or similar experiences during work**	3.15 (1.01)	3.09 (0.95)	2.84 (1.00)	< 0.001	CN = NP > HN	6
**Unfamiliarity with work, insufficient training, or not applying one’s knowledge**	2.83 (0.93)	2.81 (0.92)	2.60 (0.90)	< 0.001	CN = NP > HN	6
**Promotion stress**	2.92 (1.02)	2.70 (0.98)	2.69 (1.01)	< 0.001	CN > HN = NP	7
**Health insurance**	2.55 (1.07)	2.71 (1.08)	2.52 (1.10)	< 0.001	NP > CN = HN	8
**Lack of mental support**	2.82 (0.97)	2.90 (0.95)	2.77 (0.97)	0.003	NP > CN = HN	8

Note: The eight sources of work stress are: (1) evaluation and policy; (2) colleagues, higher-level officers, and extra-occupational duties; (3) supervisor of own department; (4) work content and opinions of individuals from other departments; (5) lack of work leadership, security, and equality; (6) impact of and fatigue from work performance; (7) promotion; and (8) others

Factors associated with hyperlipidemia, hyperglycemia, and hypertension among nurses were examined using logistic regression (Table 3). Crude odds ratio (OR) showed that NPs were 1.867-times more likely to have hyperlipidemia, hyperglycemia, and hypertension than were CNs (95% confidence interval [CI]: 1.642–2.124), and HNs were 1.501-times more likely to have hyperlipidemia, hyperglycemia, and hypertension than were CNs (95% CI: 1.262–1.784). Level of work stress correlated significantly with hyperlipidemia, hyperglycemia, and hypertension; a one-point increase in work stress generated a 1.008-times increase in risk of hyperlipidemia, hyperglycemia, and hypertension (95% CI: 1.004–1.011).

**Table 3:** Logistic regression models for factors associated with hyperlipidemia, hyperglycemia, and hypertension

	Unadjusted model OR (95% CI)	Adjusted model 1 OR (95% CI)	Adjusted model 2 OR (95% CI)	Adjusted model 3 OR (95% CI)
**Positions** (clinical registered nurses^a^)				
Nurse practitioners	1.867 (1.642–2.124)^***^	0.883 (0.738–1.057)	0.847 (0.695–1.032)	0.849 (0.696–1.035)
Head nurses	1.501 (1.262–1.784) ^***^	1.021 (0.803–1.297)	0.966 (0.743–1.258)	0.967 (0.740–1.265)
**Age (**< 26 years^a^)				
26–35	1.711 (1.406–2.081) ^***^	1.673 (1.251–2.239) ^***^	1.746 (1.271–2.399)^***^	1.759 (1.280–2.418) ^***^
36–45	4.475 (3.692–5.423) ^***^	4.125 (3.007–5.659) ^***^	4.468 (3.174–6.288) ^***^	4.605 (3.260–6.506) ^***^
46–55	11.006 (8.942–13.547) ^***^	9.856 (6.964–13.950) ^***^	11.197 (7.685–16.316) ^***^	11.718 (8.006–17.150) ^***^
> 55	19.119 (12.981–28.158) ^***^	16.361 (9.303–28.773) ^***^	25.143 (13.135–48.132) ^***^	26.149 (13.632–50.161) ^***^
**Gender (**male^a^)				
Female	1.529 (1.140–2.051) ^**^	1.442 (0.945–2.199)	1.669 (1.080–2.577) ^*^	1.674 (1.080–2.594) ^*^
**BMI** (< 18.5^a^)				
18.5–23.9	1.868 (1.467–2.378) ^***^	1.262 (0.950–1.675)	1.301 (0.948–1.786)	1.305 (0.950–1.791)
> 24	4.975 (3.904–6.341) ^***^	3.242 (2.438–4.311) ^***^	3.353 (2.439–4.612) ^***^	3.364 (2.446–4.627) ^***^
**Marital status** (never married^a^)				
Married	2.137 (1.928–2.370) ^***^	1.122 (0.947–1.330)	1.117 (0.927–1.347)	1.121 (0.930–1.352)
Divorced or widowed	2.891 (2.245–3.722) ^***^	1.203 (0.857–1.689)	1.143 (0.782–1.669)	1.140 (0.780–1.666)
**Job experience** (< 5 years^a^)				
5–9 years	2.274 (1.572–3.287) ^***^	1.296 (0.814–2.064)	1.183 (0.712–1.966)	1.208 (0.726–2.009)
10–14 years	1.698 (1.435–2.008)^***^	1.052 (0.835–1.326)	1.003 (0.778–1.292)	1.017 (0.789–1.312)
> 15 years	2.495 (2.199–2.831) ^***^	1.147 (0.947–1.390)	1.100 (0.893–1.355)	1.114 (0.904–1.374)
**Work unit** (OPD^a^)				
ER/ICU	0.599 (0.509–0.704) ^***^	0.964 (0.769–1.210)	1.004 (0.782–1.290)	1.021 (0.793–1.313)
General ward	0.618 (0.535–0.714) ^***^	0.972 (0.792–1.192)	0.968 (0.773–1.212)	0.988 (0.787–1.240)
OR/DR	0.910 (0.763–1.085)	0.981 (0.767–1.255)	0.863 (0.652–1.142)	0.866 (0.654–1.146)
Multiple units	0.890 (0.744–1.064)	0.844 (0.659–1.081)	0.815 (0.622–1.069)	0.827 (0.629–1.088)
**Weekly work hours** (≤ 40 h^a^)				
41–49 h	1.093 (0.964–1.238)	1.086 (0.928–1.270)	1.050 (0.885–1.247)	1.059 (0.892–1.258)
> 50 h	1.141 (0.995–1.307)	1.237 (1.041–1.471) ^*^	1.133 (0.937–1.370)	1.157 (0.955–1.402)
**Family care needs**	0.905 (0.822–0.996) ^*^	1.179 (1.017–1.366) ^*^	1.156 (0.982–1.360)	1.148 (0.975–1.352)
**Stress level**	1.008 (1.004–1.011) ^***^	–	1.011 (1.006–1.016) ^***^	1.011 (1.006–1.017) ^***^
**Interaction term** (NPs × stress level)	–	–	–	0.997 (0.984–1.010)
Interaction term (HNs × stress level)	–	–	–	0.999 (0.983–1.016)

^a^ Reference

*p* < 0.05^*^, *p* < 0.01^**^, *p* < 0.001^***^

Adjusted model 1 was adjusted for age, gender, marital status, work experience, work unit, weekly work hours, and family-care needs

Adjusted model 2 was adjusted for age, gender, marital status, work experience, work unit, weekly work hours, family-care needs, and stress level

Adjusted model 3 was adjusted for age, gender, marital status, work experience, work unit, weekly work hours, family-care needs, stress level, and interaction term

After controlling for other variables in the model, no significant differences were found among the nursing positions regarding the ORs for hyperlipidemia, hyperglycemia, and hypertension (adjusted model 1 and 2 in Table 3). Regarding demographic variables (adjusted model 1), participants aged > 55, 46–54, 36–45, and 26–35 years were 16.361- (95% CI: 9.303–28.773), 9.856- (95% CI: 6.964–13.950), 4.125- (95% CI: 3.007–5.659), and 1.673-times (95% CI: 1.251–2.239), respectively, more likely to have hyperlipidemia, hyperglycemia, and hypertension than those aged under 26 years. Nurses with a BMI of > 24 kg/m^2^ were 1.237-times (95% CI: 1.041–1.471) more likely to have hyperlipidemia, hyperglycemia, and hypertension than those with a BMI of < 24 kg/m^2^. Nurses who worked over 50 h a week were 1.237-times (95% CI: 1.041–1.471) more likely to have hyperlipidemia, hyperglycemia, and hypertension than those who worked 40–49 h a week. Finally, nurses who had family-care needs were 1.179-times (95% CI: 1.017–1.366) more likely to have hyperlipidemia, hyperglycemia, and hypertension than those without family-care needs.

In adjusted model 2, we added level of work stress to adjusted model 1. Level of work stress was significantly associated with hyperlipidemia, hyperglycemia, and hypertension; every one-point increase in work stress corresponded to a 1.011-times increase in the OR of hyperlipidemia, hyperglycemia, and hypertension (95% CI: 1.006–1.016). After adding level of work stress, both weekly work hours and family-care needs lost their significance. These results suggested that the effect of extended work hours (≥ 50 h) and family-care needs on hyperlipidemia, hyperglycemia, and hypertension can be explained by perceived level of work stress. After adding level of work stress to the model, we found that women were 1.669-times (95% CI: 1.080–2.577) more likely to have hyperlipidemia, hyperglycemia, and hypertension than men. Age and BMI remained risk factors for hyperlipidemia, hyperglycemia, and hypertension. The results of adjusted model 3 showed that there was no significant interaction between job position and level of work stress in regard to hyperlipidemia, hyperglycemia, and hypertension, meaning the association between work stress and hyperlipidemia, hyperglycemia, and hypertension did not differ among HNs, NPs, and CNs.

We further used logistic regression to analyze whether the association between work stress and hyperlipidemia, hyperglycemia, and hypertension differed across sources of work stress (Table 4). The adjusted model (Table 4) showed that all eight sources of stress were significantly associated with hyperlipidemia, hyperglycemia, and hypertension. Their CIs overlapped, indicating that the association between work stress and hyperlipidemia, hyperglycemia, and hypertension did not significantly differ across the sources of work stress.

**Table 4:** Logistic regression results for the effect of work stress from different sources on hyperlipidemia, hyperglycemia, and hypertension

Source of stress	OR (95% CI)
Adjusted model (aspects 1)	1.083 (1.048–1.118)^***^
Adjusted model (aspects 2)	1.054 (1.030–1.079) ^***^
Adjusted model (aspects 3)	1.117 (1.040–1.200) ^***^
Adjusted model (aspects 4)	1.040 (1.016–1.064) ^***^
Adjusted model (aspects 5)	1.054 (1.028–1.081) ^***^
Adjusted model (aspects 6)	1.032 (1.016–1.049) ^***^
Adjusted model (aspects 7)	1.104 (1.036–1.177) ^***^
Adjusted model (aspects 8)	1.083 (1.042–1.126) ^***^

Note: Eight aspects: (1) evaluation and policy, (2) colleagues, higher-level officers, and extra-occupational duties, (3) supervisor of own department, (4) opinions of individuals from other departments, (5) lack of work leadership, security, and equality, (6) impact of and fatigue from work execution, (7) promotion, (8) others

Model was adjusted for age, gender, job positions, marital status, work experience, work unit, weekly work hours, and family care needs

## Discussion

The present research found significant differences among HNs, NPs, and CNs regarding sources of perceived work stress; these differences in stressors were determined to be associated with the different work contents of the respective job positions. Our results showed that evaluation, hospital management, and communication with others is the primary stressor for HNs, that work complexity, uncertainty regarding medical results, urgency of work, and lack of job security and support is the primary stressor for NPs, and that promotion is the primary stressor for CNs. Hospitals in Taiwan must undergo evaluations every three years to certify/retain their status as medical centers, regional hospitals, district hospitals, and/or teaching hospitals. In addition, there are several other types of external evaluations/certifications that hospitals can seek to underline their high standards and quality of care [30]. HNs are the head administrators in departments of nursing, and are usually the leaders of unit evaluations; this explains why evaluation and management-related aspects represented the primary stressor for this group [31]. On the other hand, NPs and CPs experience most stress from heavy workloads or extended working hours, which may be due to their engagement in shift work and workforce shortages. Workforce shortages could increase nurses’ working hours, reduce some of their off hours, induced burnout, and exacerbate workplace bullying [32]. Further, workforce shortages and heavy workloads in the nursing sector have become core issues in Taiwan’s national policies. Although existing policies in Taiwan stipulate that nurse–patient ratios should not exceed 1:9 in medical centers, 1:12 in regional hospitals, and 1:15 in district hospitals [33], these ratios are still higher than the corresponding ratios in many developed countries, such as the US, Japan, and European countries [34, 35]. An excessive nurse-patient ratio may cause occupational burnout and turnover intentions as well as an increased risk of medication errors and a growth in patient mortality rates, ulcers, infection, and pneumonia [32, 34, 36]. Thus, when drafting stress-management solutions for nurses, hospitals should consider the diverse influences of different sources of stress and job positions.

Although the significance of each source of stress varied across the different nursing positions, the overall work-stress perception did not significantly differ across the sample. The prevalence of hyperlipidemia, hyperglycemia, and hypertension differed across job positions, being highest among HNs, followed by NPs and CNs. Through univariate analysis, we observed distributional differences among the three nursing positions in terms of age, gender, BMI, marital status, years of work experience, work unit, work hours per week, and family-care needs. In sum, nurses in all three job positions had distinct characteristics, and these attributes, rather than job position, influenced prevalence of hyperlipidemia, hyperglycemia, and hypertension.

Our finding regarding factors related to hyperlipidemia, hyperglycemia, and hypertension agree with previous findings. Older individuals have a higher risk of suffering from hyperlipidemia, hyperglycemia, and hypertension [9, 37]. Excessive BMI also has a significant correlation with hyperlipidemia, hyperglycemia, and hypertension [36, 38]; the present study defined obesity as BMI > 24 in accordance with the criteria in Taiwan; among the participants, 33.0% of HNs were obese, followed by NPs (26.7%) and CNs (25.2%). The HNs’ average age was higher than those of the other groups, and they mainly performed administrative work. Further, most NPs and CNs worked on the front line, which entails more physically strenuous tasks. Regarding the correlation of hyperlipidemia, hyperglycemia, and hypertension with working hours, the longer the working hours, the higher the risk of developing hyperlipidemia, hyperglycemia, and hypertension [39]. Finally, regarding the finding that participants who had family-care needs also showed a higher risk of hyperlipidemia, hyperglycemia, and hypertension, previous research has reported that individuals with family-care needs have a stronger stress perception than do those without such needs [40], which would further impact hyperlipidemia, hyperglycemia, and hypertension [41].

Our study revealed that an increased level of perceived stress is associated with an increased OR for hyperlipidemia, hyperglycemia, and hypertension, which is consistent with the literature [3–5]. Stress perception is likely to lead to the adoption of harmful lifestyles, such as smoking, alcoholism, high-sugar and high-fat diets, and insufficient exercise [12, 42]. Without early prevention, job stress and an unhealthy lifestyle can not only increase the risk of hyperlipidemia, hyperglycemia, and hypertension, but also lead to cardiovascular diseases [8, 4]. Our results suggest that nursing management in hospitals should utilize appropriate stress management strategies and plan stress reducing interventions. There is a need for effective stress relieving and health enhancing lifestyle programs for nurses in hospitals.

### Limitations

This study used self-report data, and we were unable to verify the accuracy of the participants’ reports. No objective measurement of hyperlipidemia, hyperglycemia, and hypertension was available, and under-reporting was possible; however, the rate of under-reporting across the three groups could be non-differential. Finally, causal relationships could not be inferred as a result of the use of a cross-sectional design.

## Conclusions

Medium to high job stress was observed in nurses. Although stressors varied by nursing position, the overall stress level was similar across HNs, NPs, and CNs. Higher job stress levels, increased age, higher BMI, long work hours, and caring for family members were positively associated with hyperlipidemia, hyperglycemia, and hypertension. Further studies are needed to identify and verify effective strategies for reducing work stress in nursing staff.

## Data Availability

The data that support the findings of this study are available from the Health Promotion Administration, Taiwan. Restrictions apply to the availability of these data, which were used under license for this study. Data are available with the permission of the Health Promotion Administration, Taiwan.

## Abbreviations

US: United States
NP: Certified nurse practitioner
CN: Clinical registered nurse
HN: Head nurse/ nurse manager
ANOVA: Analysis of variance
SD: Standard deviation
BMI: Body mass index
OR: Odds ratio
CI: Confidence interval
DR: Delivery room
ER: Emergency room
ICU: Intensive care unit
OPD: Outpatient clinic
OR: Operating room
